# Long-term results of the Burch-Schneider antiprotrusio cage: a single-centre follow-up of 144 cases after a minimum of 5 years

**DOI:** 10.1177/11207000251362177

**Published:** 2025-08-18

**Authors:** Thomas Stark, Karl Stoffel, Thomas Ilchmann, Brigitta Gahl, Lukas Zwicky, Peter E Ochsner, Martin Clauss

**Affiliations:** 1 Department of Orthopaedics and Trauma Surgery, University Hospital Basel, Basel, Switzerland; 2 Department of Orthopaedics, Hirslanden Klinik Birshof, Munchenstein, Switzerland; 3 University Hospital Basel - Surgical Outcome and Research Centre (SORC), Basel, Switzerland; 4 Department of Orthopaedics and Trauma Surgery, Kantonsspital Baselland, Liestal, Switzerland; 5 Department of Orthopaedics and Trauma Surgery, Centre for Musculoskeletal Infections (ZMSI), University Hospital Basel, Basel, Switzerland

**Keywords:** Burch-Schneider antiprotrusio cage, hip, long-term results, major acetabular bone defects, revision total hip arthroplasty

## Abstract

**Background:**

Although the Burch-Schneider antiprotrusio cage (BS-APC) has been reported to be reliable, long-term data for this implant are scarce. We thus aimed to investigate survival and radiological results for revision total hip arthroplasty with the BS-APC in patients with major bone deficiency (55% AAOS defect grade 3, 39% grade 4) who had a minimum follow-up of 5 years (mean 10.2 years).

**Methods:**

144 revisions in 140 patients were performed due to aseptic loosening (*n* *=* 74), infection (*n* = 50), or other reasons (*n* = 20). Survival analysis was performed with death as a competing risk. Clinical follow-up was performed at 1, 2, and 5 years and every 5 years thereafter.

**Results:**

77 patients died during follow-up, 25 within the first 5 years. 12 BS-APCs were re-revised for infection (*n* = 5), aseptic loosening (*n* = 5), or instability (*n* = 2). The cumulative incidence for aseptic re-revision of BS-APCs was 4.3% at 10 years (95% CI, 1.8–10.1%), and the cumulative risk of death was 73.3% (95% CI, 62.4–83.2%). Radiological changes occurred in 26 of 87 radiologically examined hips, of which 8 cases were revised.

**Conclusions:**

We found excellent mid- and long-term survival of the BS-APC in acetabular revision with major bone deficiencies, in accordance with or superior to most literature reports, which might be explained by strict adherence to surgical technique.

## Introduction

As the age for patients undergoing total hip arthroplasty (THA) becomes lower and life expectancy increases, the number of revision THAs (rTHAs) inevitably increases.^1^ With repeated revisions, bony defects get bigger, especially on the acetabular side, resulting in inferior long-term results compared with those after primary surgery and revision surgery with small defects.^2^ Filling large acetabular defects with bone grafts that lack support results in high failure rates caused by graft resorption and loosening.^3^ Historically, this led to the development of metal meshes to support bone impaction grafting, which is technically demanding but shows excellent long-term survival and offers biological restoration of the bone for further revisions.^4^ For smaller acetabular defects, acetabular reconstruction rings such as the Mueller acetabular reinforcement ring (ARR) have shown excellent long-term survival.^5^ When bony defects are larger, a reconstruction system that bridges bone defects is recommended to safeguard the graft and ensure structural stability,^6^ especially when the graft supports over 50% of the acetabular component.^7^ An alternative method to address large bone defects is to fill them with metal implants such as jumbo cups,^8^ augments,^9^ which may be combined with porous metal cups,^10^ cup and cage constructs,^11^ or custom-made triflange acetabular implants.^12^ This approach is technically less demanding but results in even larger defects in the case of re-revision.

In our department, extensive acetabular bone defects have been managed using the Burch-Schneider antiprotrusio cage (BS-APC), with or without the addition of morselized bone impaction grafting.^13^ Although various studies have shown the implant to be reliable, mid- to long-term data for this device are still scarce.^14,15^ We therefore investigated long-term survival and radiological results for rTHA with major acetabular defects with the use of the BS-APC.

## Patients and methods

The study was conducted in accordance with the Declaration of Helsinki and was approved by the Ethikkommission Nordwest- und Zentralschweiz (BASEC ID 2017-01931).

Between October 1988 and June 2012, a total of 608 acetabular revisions were performed in our department.

During the observation period the majority of acetabular revision (66%) in our institution were performed using an ARR, in rare cases either primary pressfit cups or cement all poly cups were used.^5^ In 144 revision procedures involving 140 patients (74 females, 66 males) (Table 1), a BS-APC was used for acetabular revision after assessment of the acetabular defect.13 The median age at the time of revisions was 72 years (range 22–94 years). In 67 cases, the right hip was operated. The indication for cup revision was aseptic loosening in 74 cups (51%), periprosthetic joint infection (PJI) in 50 cups (35%), and other reasons in 20 cups (14%). The type and extent of acetabular defects were classified according to the American Academy of Orthopaedic Surgeons (AAOS) described by D’Antonio et al.^16^ with the help of preoperative anteroposterior pelvic radiographs and descriptions from the operative reports. During the study period preoperative CT scans were seldom obtained, therefore information from these scans were not included in the analyses presented in this study. Additional stem revision was performed in 80 cases. Femoral stems were revised based on intraoperative assessment of stability, with loose or infected stems being replaced. Implant selection was determined by surgeon preference and extent of femoral bone loss and comprised mainly cemented and uncemented revision stems. Details on stem selection and follow-up has recently been published in detail.^17,18^

**Table 1.** Demographic and clinical data of 140 patients (144 hips).

Characteristic^a^	
Age, years	72.3 (21.6–93.5)
Male:female ratio	66:74
BS-APC type and size	
Gen I (*n* = 49)^b^	
Size 44	15
Size 50	30
Size 56	4
Gen II (*n* = 24)^c^	
Size 44	2
Size 50	21
Size 56	1
Gen III (*n* = 71)^d^	
Size 44	5
Size 50	66
Inlay	
Metasul	4
Polyethylene	139
seleXys DS	1
Grafts^e^	
Cemented	12
Bone	82
No graft	24
unknown	26
AAOS classification	
2	4
3	70
4	60
N.A.	10

BS-APC, Burch-Schneider antiprotrusio cage; N.A., not applicable; AAOS, American Academy of Orthopaedic Surgeons.

a All values are given as raw numbers, except for age, which is given as mean (range).

b The Gen I design of BS-APC corresponds to a design without additional holes in the ischial flange.

c Gen II is a smoothly blasted design.

d Gen II is further advanced by Gen III, a newer roughly blasted cage.

e Graft options included homologous, autologous from acetabulum or iliac crest, and bovine Tutobone.

Survival analysis of the BS-APC was performed, with death of the patient as a competing risk. Clinical evaluation by means of the Harris Hip Score and pain levels, along with radiological follow-ups, adhered to our institutional protocol. These evaluations were conducted at intervals of 1, 2, and 5 years and every 5 years thereafter. Eight patients (9.7%) were lost to follow-up prior to 5 years, with an additional 25 patients (16.7%) lost due to death before their examination 5 years after implantation of the BS-APC (Table 2). Anteroposterior radiographs of the pelvis were assessed right after implantation of the antiprotrusio cage, before re-revision of the BS-APC or in hips with a minimum of 5 years follow-up.

**Table 2.** Patient and follow-up characteristics in 144 hips (140 patients): revisions and mortality overview.

Follow-up	Patients	Hips
Within 5 years		
Total number	140	144
Mortality	25	
Lost to 5-year follow-up	8	
Revisions		4
Later than 5 years		
Total number	104	107
Mortality	52	
Revisions		8
Lost to follow-up	8	
Available x-ray		87

Radiographs were evaluated for osteolysis, migration of the BS-APC, and loosening. Furthermore, the location of the caudal flange inside the ischial bone was screened at the first and final radiograph. Osteolysis was defined as progressive bone resorption ⩾2 mm within the zones specified by DeLee and Charnley,^19,20^ which was absent on the initial postoperative radiograph.^21^ Loosening criteria included breakage of over 50% of screws, progressive radiolucent lines around the implant or 50% of screws.^5,18^ Migration was assessed by measuring the vertical displacement of the cup centre relative to the inter-teardrop line and the horizontal displacement of the cup centre concerning the ipsilateral teardrop line, as described by Nunn et al.^22^ A change in the cup inclination angle of more than 4° and vertical and/or horizontal displacement >3 mm was taken as evidence of a loose cup.^23^ Allograft osteointegration was confirmed by trabecular bridging, normal graft density, absence of fragmentation,^24^ and absence of radiolucent lines.^25^ Bone resorption was identified as density reduction or breakdown of the transplanted bone.^26^

### Implants

The BS-APC that was developed in 1974 was thought to be similar to a bridging plate, connecting the native bone over the acetabular defect to allow bone graft integration.^27^ Initial screw fixation should be oriented in the direction of the iliosacral joint; the ischial flange was designed to be inserted into the ischial bone to bridge extended acetabular bone defects and acetabular protrusion.^28^

### Surgical technique

**Figure 1.**
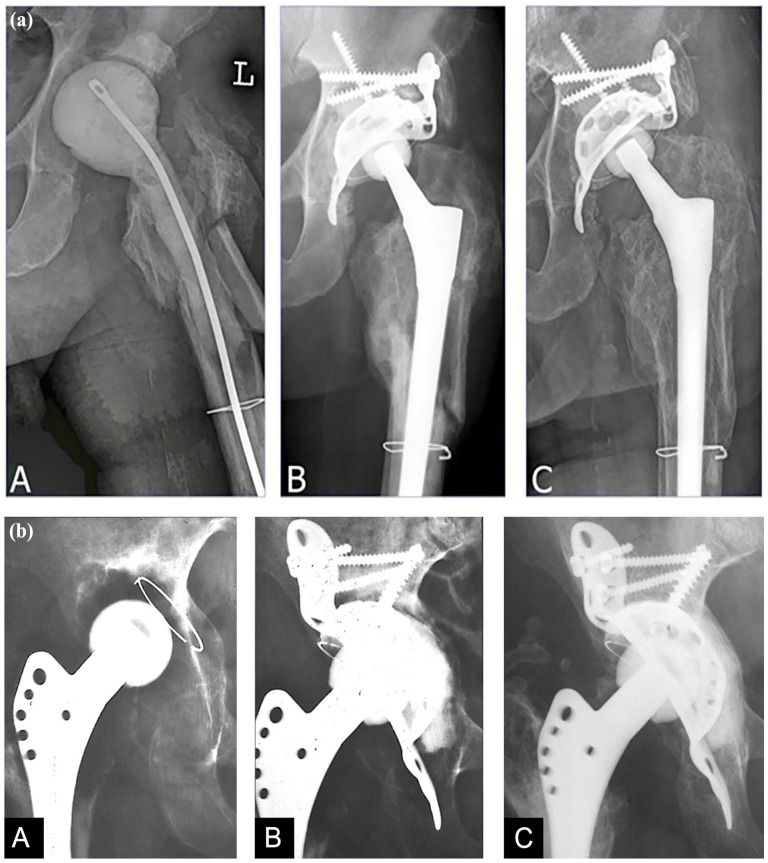
(a) Case description of a 76-year-old male patient. (A) 2-stage revision for periprosthetic joint infection with a cement spacer implanted. Intraoperative acetabular defect size AAOS 3. (B) Postoperative radiograph of implanted Burch-Schneider antiprotrusio cage, autologous bone graft, and femoral stem. Long screw pointing toward the iliosacral joint placed first. (C) Follow-up 11.2 years after revision showing no signs of loosening. (b) Case description of a 59-year-old female patient. (A) Massive loosening of a conic Endler polyethylene cup. Intraoperative acetabular defect size AAOS grade 4. (B) Following curettage, morsellised iliac crest autograft was placed cranially and medially in the cavity, and an BS-APC was implanted (C) Complete osseous integration without loosening or wear at 11-year follow-up.

In all cases, a transgluteal Hardinge approach with patients in the supine position was performed, using the previous incision. After removal of the loose cup, thorough debridement to expose vital acetabular bone was performed with drills and curettes.^17^ Thereafter, the outer ilium wall above the defect was cleared from soft tissue. The remaining bony deficiency was assessed, and the BS-APC was customised by using trial cages to estimate the bending of proximal and distal flanges to fit the acetabular cavity and surrounding bone. The ischial bone was opened with a chisel and widened with a clamp to allow flange insertion. Autologous grafts were used to fill weight-bearing defects, while deep-frozen allografts were applied in non-weight-bearing areas or elderly patients; a cement pillar was occasionally added craniomedially for enhanced primary stability.^13^ Afterward, the BS-APC was inserted. Screw fixation always started with 6.5-mm cancellous bone screws from inside the cage directed toward the iliosacral joint (Figure 1), centring the cage inside the acetabulum, compressing the graft, and promoting angular stability after cementing.^27^ The proximal flange was fixed to the iliac bone sidewall with two to four 6.5-mm cancellous screws. Finally, polyethylene cups were securely fixed with bone cement (Palacos R+G, Heraeus) at angles of 40–45° inclination and 15° anteversion, without the use of jet lavage or cement pressurising before cup insertion. Patients were mobilised with full weight-bearing as tolerated from the first postoperative day by using 2 crutches or other aids if needed.

### Radiological follow-up

Throughout follow-up, standardised anterioposterior radiographs were taken, centred on the symphysis and capturing the entire prosthesis. Digitised images from the last preoperative radiograph, the immediate postoperative period, and the latest follow-up were examined with DICOM software (Agfa IMPAX v6.5.3.117; Agfa HealthCare, Mortsel, Belgium) by calibrating against the true femoral head size. Measurements were performed with AGFA-Orthopaedic-Tools (Version 2.10 [Build 4]; Agfa HealthCare). Acetabular defects were preoperatively classified according to the AAOS system.^16^ Postoperatively, radiographs were examined for osteolysis, signs of loosening, and migration.

### Statistics

We derived cause-specific cumulative incidence rates of revision, modeling death as a competing event, as determined by the Kaplan-Meier score.^29^ We established the 95% confidence interval (CI) of the survivorship curves with Greenwood’s formula. We then calculated cause-specific cumulative incidence rates of revision for aseptic loosening, modelling revision for other indications, and death as competing events. Lastly, we constructed a worst-case scenario, assuming that each right-censored patient – and hence each patient without an observed event – experienced revision for aseptic loosening immediately after the end of follow-up. We used Stata 16.0 (StataCorp LLC, 4905 Lakeway drive, College Station, Texas) for conducting the analysis.

## Results

### Clinical follow-up

**Figure 2.**
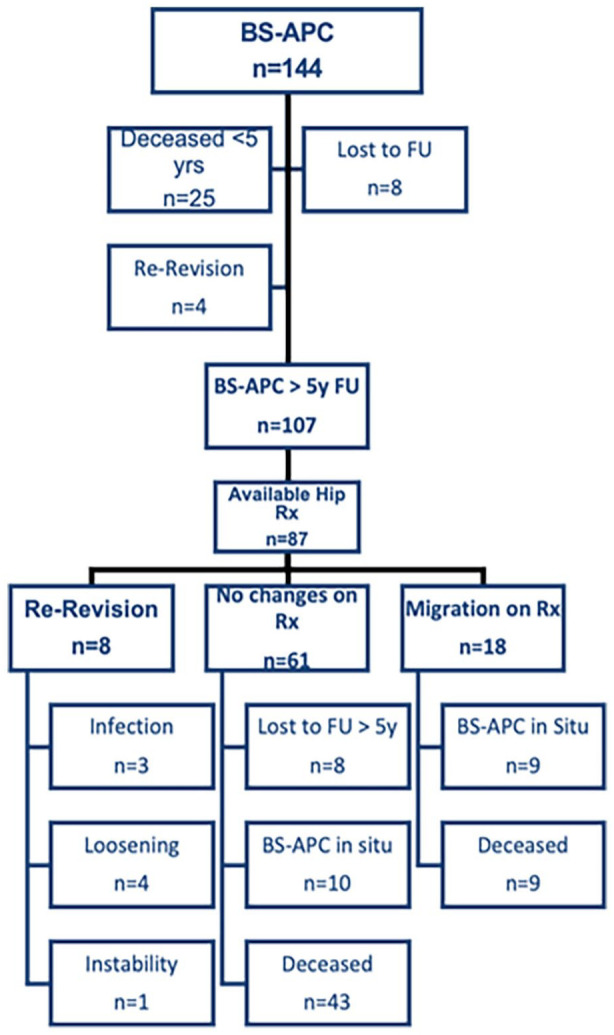
Flow chart highlighting survival status at last follow-up (FU) and available radiographs (Rx) for analysis. BS-APC, Burch-Schneider antiprotrusio cage.

The mean follow-up time was 10.2 years (5–23.4 years). 77 patients (55%) died for causes not related to the revision during follow-up, 25 (17.4%) of them within the first 5 years. 16 patients (11.1%) were lost to follow-up during the study period, 8 (50%) of them within the first 5 years (Figure 2).

Of 18 patients who had a follow-up <5 years, 10 could be contacted and confirmed that their implants were in place but refused to be examined due to absence of symptoms. 8 either moved and could not be traced or were assumed to be dead due to old age at the time of analysis or known unfavourable prognosis of underlying disease. At final follow-up, 12 BS-APCs were re-revised after an average of 5.0 (1.1–11.4) years: 5 due to periprosthetic infection (2 of which had recurrent infection), 5 due to aseptic loosening, and 2 due to malpositioning of the cup, causing instability and repeated dislocation. In one case of re-revision due to aseptic loosening, the cup and the inserted screws broke. Of the 12 re-revisions, 4 took place within 5 years of implantation, 6 between 5 and 10 years, and 2 after 10 years of follow-up. None of the 5 BS-APCs that were followed up for more than 15 years needed revision.

A total of 79 (55%) of the acetabular defects were classified as AAOS defect grade 3 with segmental and cavitary deficiencies and 65 (39%) as grade 4 with pelvic discontinuity. The caudal flange was inserted into the ischium in 105 cases (73%). The grafting material used for filling the acetabular defect is shown in Table 1. The mean Harris Hip Score final follow-up was 78 (range 21–96). Of the patients, 88% had no or mild pain, and 12% reported moderate hip pain.

### Radiographic analysis

87 BS-APCs were analysed with a mean follow-up of 10.2 years (5–23.4 years), among which 61 cases (71%) showed no radiological changes during follow-up. The remaining 26 cases had a median follow-up of 8.2 years (range 4.7–15.1 years). Eight were revised for infection (*n* = 3), aseptic loosening (*n* = 4), or malpositioning (*n* = 1). The remaining 18 patients showed signs of implant migration (>3 mm), 9 of whom died during further follow-up at an average of 9.3 years (range 5.1–14.4 years) without the implant being revised or being symptomatic for aseptic loosening. Of the remaining 9 patients with detectable migration, none was scheduled for revision at a mean of 10.6 years (range 4.9–15.1 years). Osteolysis was found in 5 cases, and in 4, the used bone graft had no signs of bony incorporation or was fragmented. The classification of preoperative acetabular defects, as well as radiographic changes in 26 of 87 examined BS-APCs, are highlighted in Table 3.

**Table 3.** Reasons for re-revision of the BS-APC and radiological studies confirming migration of the construct above the threshold.

Reason		BS-APC survival	Gender	Age (years)	BMI (kg/m^2^)	Type of implant	AAOS class	Radiological changes			
								Inclination	X-axis	Y-axis	Screw breakage
Revised											
	Infection										
		1.26	m	69.5	27.4	Rough	3		5.38	−3.57	
		1.13	m	35.5	24.7	Rough	3	−5.54	3.02		
		5.57	m	76.6	26.8	Rough	4				
		7.94	f	75.4	33.6	No holes	4	−8.78	6.42	−3.53	
		9.65	f	63.9	20.5	No holes	4		8.63	31.24	x
	Aseptic loosening										
		2.39	f	60.6	25.6	Rough	4	−4.56			
		4.65	f	78.3	18.1	Smooth	4		−3.29	3.58	
		5.51	f	62.3	18.4	No holes	3				
		8.86	f	46.1	23.7	Rough	3	−32.13	16.04	10.75	
		11.39	m	57.3	24.2	Rough	3			9.23	
	Malposition										
		1.21	f	42.1	19.1	No holes	3	−15.74		−5.18	
		5.27	m	78.3	33.6	Rough	4	−9.17	7.59	7.5	x
Non-revised											
	BS-APC *in situ*										
		5.68	f	65.5	29.7	Rough	4	8.80	−4.79		
		10.04	f	69.3	33.7	Rough	3	−4.1			
		4.94	m	63.5	28.1	No holes	4		−3.2		
		10.04	m	71.4	24.0	Rough	4		−3.1	4.5	
		14.37	m	75.6	27.0	Rough	3		−3.9		x
		14.74	f	65.0	46.4	Rough	3		−3.4		
		5.92	f	82.7	25.7	Smooth	3			4.1	
		14.29	m	72.3	32.1	Rough	3			−3.7	
		15.06	m	67.1	33.6	Rough	3			4.1	
	Death after 5 years										
		14.44	f	73.3	31.6	Rough	3	5.45	8.24	−8.6	
		5.11	f	83.2	27.5	Rough	3		10.2		
		6.08	f	81.8	na	Rough	4		−3.9	4.6	x
		11.92	m	81.5	24.3	Smooth	3		−3.7		
		13.07	m	81.6	25.1	Smooth	4		−3.5	3.3	
		6.39	f	87.4	22.0	No holes	3			−10.7	
		6.53	f	86.7	23.7	Rough	3			3.8	
		7.96	m	81.6	27.0	No holes	4			3.4	x
		12.28	m	83.5	22.0	Smooth	4			6.6	

BS-APC, Burch-Schneider antiprotrusio cage; BMI, body mass index; AAOS, American Academy of Orthopaedic Surgeons; m, male; f, female; na, not available.

Note: In 9 cases, the BS-APC was still in place at last follow-up, without further revision planned. In another 9 cases, migration was detected radiologically, but the patients died before further intervention became necessary.

### Survival analysis

**Figure 3.**
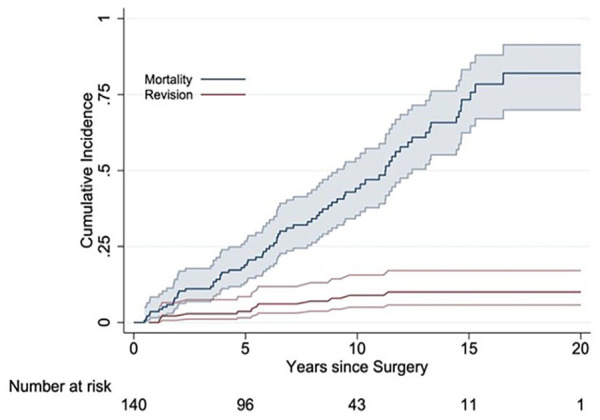
Cumulative risk for reoperation for aseptic loosening of the Burch-Schneider antiprotrusio cage (BS-APC) is shown at approximately 5 years, 10 years, and 15 years (red line). Cumulative risk of death (blue line [upper line]) for patients who underwent revision surgery with the BS-APC is shown at 5 years, 10 years, and 15 years.

The cumulative risk for reoperation (CRR) for aseptic loosening of the BS-APC was approximately 3.2% at 5 years and 4.3% at 10 years, with the incidence for re-revision for any reason being 8.9% at 5 years and 10% at 10 years (Table 3). In a worst-case scenario, with all BS-APCs lost to follow-up that were expected to be revised for aseptic loosening, the resulting CRR would be 31.6% at 5 years, 42.2% at 10 years, and 45.7% at 15 years. Analysis incorporating death as a competing risk demonstrated that the probability of revision for any reason was substantially lower than the probability of death over the observed period. The cumulative risk of death for patients who underwent revision surgery with the BS-APC was approximately 18.9% within 5 years, 44.1% between 5 and 10 years, 73.3% between 10 and 15 years, and 82.0% after 15 years. Survival of the BS-APC with removal for aseptic loosening or radiographic failure as the end points was 84.7% at 10 years (Figure 3).

## Discussion

Our study presents the outcomes following the use of BS-APC exclusively as a revision implant for acetabular defects categorised as AAOS 3 and 4. In the literature, survival data for the BS-APC are conflicting, ranging from 62% to 90.6%.^4^ Long-term survival for the BS-APCs in our series is excellent, with a CRR of 10.0% for revision for any reason as the endpoint.

### Aseptic loosening

5 out of 144 cases (3,5%) were revised for aseptic loosening which was the most frequent cause of failure in our series. Implant stability depends on surgical experience and operative technique. The inventors described 3 important steps for inserting the BS-APC:^13,27^ first, fill defects with morsellised autografts and allografts; second, compress the graft and the implant against the acetabulum, starting with screws directed toward the iliosacral joint, centring the cage inside the acetabulum; and third, insert the iliac flange inside the acetabulum to neutralise forces levering out the BS-APC supero-laterally. An essential step for achieving primary stability of the BS-APC lies in applying pressure of the cage against the remaining acetabular roof and the bone graft by using cranio-caudo-medial screws through the cage. By vigorously using this technique, we found no case of graft resorption in the whole series and excellent restructuring of the implanted grafts, which can also be seen in other series.^3,30^ A side effect from using the screws in that way is additional angular stability of these screws due to fixation of the screw heads with the cement used to fix the cup.^13^ Finally, inserting the ischial flange of the BS-APC cage into the ischial bone is crucial to prevent lateralisation of the construct and maintain the restored centre of rotation,^30,31^ thereby preventing complications such as mechanical failure, leg-length discrepancy, and loosening.^13^

Aseptic loosening of a cup starts with cup migration. Even with a threshold of 3 mm for detectable migration of the cup^22^ instead of 5 mm,^30^ the detected mid- to long-term migration rate in our series was low (4.3%) compared to that reported in the literature (0–28%).^4,15,31^ Micromotion of the BS-APC is well documented,^32^ in our series all cases with aseptic loosening showed supero-lateral migration which is a well-established mode of failure in literature.^33^ While the BS-APC is designed to resist medial migration compressing and protecting the bone graft and providing structural support, they do not inherently restrict lateral displacement, especially with the flange not inserted into the ischium.

### Periprosthetic infection (PJI)

5 (3.5%) cases had to be re-revised due to PJI. 2 PJI were due to persistent infection after 50 cases of PJI included in this series and required re-revision with a new 2-stage revision after 1.1 years and 1.3 years follow-up. 3 cases showed late haematogenous PJI after 5.6, 7.7 and 9.7 years after aseptic revision. All had a 2-stage revision. Infection is a leading cause of failure in revision arthroplasty.^18,32^

### Orientation of the cup and instability

Modern highly porous-coated hemispherical cups with augments or cup-cage constructs made out of novel materials such as tantalum hold the potential for superior long-term efficacy compared with conventional antiprotrusio cages, as they facilitate bony ingrowth, thereby enhancing stability.^34^ The drawback of these implants is that they rely on primary press-fit fixation, which might compromise implant orientation. We found a very low dislocation rate (2 cases, 1.4%) in our cohort by using 28-mm heads compared to recently reported rates of 3.3–7.4%.^4^ A potential explanation might be the freedom of orientation of the polyethylene cup inside the fixed cage. After reconstructing the acetabular anatomy with the BS-APC cup, orientation can be optimised separately.^13^

There are several limitations with our cohort that should be addressed: the high mortality rate observed in our cohort (55%) is in line with previously published data and reflects the complexity of the procedures, the advanced age of the patients, and their associated comorbidities.^18^ Notably, 25 patients (17.9%) died within the first 5 postoperative years. Among the deceased patients, 43 (30.1%) showed no radiological signs of implant migration at their last follow-up, while 9 (6.4%) exhibited migration but were not scheduled for re-revision. Although undocumented revisions at other institutions cannot be fully excluded, this is considered unlikely given that the majority of patients were elderly and local, typically preferring to receive care at our centre.^17^

8 patients were lost to follow-up after 5 years, including 6 untraceable individuals residing elsewhere. In the worst-case scenario, assuming all lost cases were revised due to aseptic loosening, the cumulative incidence rates for reoperation for the BS-APC at the 20-year mark would be 10.9% (95% CI, 5.0–16.7%), still indicating excellent outcomes.

## Conclusion

We found excellent mid- and long-term survival for the BS-APC in acetabular revision with major bone defects. Our results were in accordance with or even better than reports in the literature, which might be explained by strict adherence to the surgical technique described for the BS-APC.
